# Diagnosis of Bladder Cancer Recurrence Based on Urinary Levels of *EOMES*, *HOXA9*, *POU4F2*, *TWIST1*, *VIM,* and *ZNF154* Hypermethylation

**DOI:** 10.1371/journal.pone.0046297

**Published:** 2012-10-03

**Authors:** Thomas Reinert, Michael Borre, Anders Christiansen, Gregers G. Hermann, Torben F. Ørntoft, Lars Dyrskjøt

**Affiliations:** 1 Department of Molecular Medicine, Aarhus University Hospital, Aarhus, Denmark; 2 Department of Urology, Aarhus University Hospital, Aarhus, Denmark; 3 Department of Urology, Frederiksberg Hospital, Copenhagen University, Frederiksberg, Denmark; Geisel School of Medicine at Dartmouth, United States of America

## Abstract

**Background:**

Non muscle invasive bladder cancer (NMIBC) has the highest recurrence rate of any malignancy and as many as 70% of patients experience relapse. Aberrant DNA methylation is present in all bladder tumors and can be detected in urine specimens. Previous studies have identified DNA methylation markers that showed significant diagnostic value. We evaluated the significance of the biomarkers for early detection of tumor recurrence in urine.

**Methodology/Principal Findings:**

The methylation levels of *EOMES*, *HOXA9*, *POU4F2*, *TWIST1*, *VIM,* and *ZNF154* in urine specimens were measured by real-time PCR (MethyLight). We analyzed 390 urine sediments from 184 patients diagnosed with NMIBC. Urine from 35 age-matched control individuals was used to determine the methylation baseline levels. Recurrence was diagnosed by cystoscopy and verified by histology. Initially, we compared urine from bladder cancer patients and healthy individuals and detected significant hypermethylation of all six markers (P<0.0001) achieving sensitivity in the range 82%–89% and specificity in the range 94%–100%. Following, we validated the urinary hypermethylation for use in recurrence surveillance and found sensitivities of 88–94% and specificities of 43–67%. *EOMES*, *POU4F2*, *VIM* and *ZNF154* were more frequently methylated in urine from patients with higher grade tumors (P≤0.08). Univariate Cox regression analysis showed that five markers were significantly associated with disease recurrence; *HOXA9* (HR = 7.8, P = 0.006), *POU4F2* (HR = 8.5, P = 0.001), *TWIST1* (HR = 12.0, P = 0.015), *VIM* (HR = 8.0, P = 0.001), and *ZNF154* (HR = 13.9, P<0.001). Interestingly, for one group of patients (n = 15) we found that hypermethylation was consistently present in the urine samples despite the lack of tumor recurrences, indicating the presence of a field defect.

**Conclusion/Significance:**

Methylation levels of *EOMES*, *HOXA9*, *POU4F2*, *TWIST1*, *VIM,* and *ZNF154* in urine specimens are promising diagnostic biomarkers for bladder cancer recurrence surveillance.

## Introduction

Cancer of the urinary bladder is the fifth most common neoplasm in the industrialized countries. In approximately 75% of all cases, the patients will present with stage Ta or T1 non-muscle invasive bladder cancer (NMIBC), whereas the remaining 25% of the tumors will be muscle invasive stage T2-4 cancers (MIBC) [1]. About 60%–70% of patients with NMIBC experience tumor recurrences within 3 years after resection [2], [3] and patients may develop recurrent tumors annually for many years without disease progression. However, up to 25% will progress to muscle invasive disease [4]. The high recurrence rate and the risk of progression prompt the need for frequent and long surveillance, making bladder cancer the most expensive cancer to treat [5], [6]. Following resection of the primary tumor, patients are frequently monitored by cystoscopy and cytology. Biopsies taken during cystoscopy are the gold standard for diagnosing bladder tumors. The sensitivity of cystoscopy for NMIBC is close to 80% for white light cystoscopy, and 96% when using the more costly, fluorescence-guided cystoscopy using hexaminolevulinate (HAL). For detection of dysplasia and carcinoma in situ (CIS), the sensitivity of white light cystoscopy decreases to 48% and 68%, respectively; whereas the sensitivity of cystoscopy using HAL for these lesions remains in the range from 93%–95% [7]–[9]. Cytology is often used in combination with cystoscopy, owing to a high specificity of 99% (0.83–0.997; 95% CI), but with a low sensitivity of 34% (0.20–0.53; 95% CI). The sensitivity of cytology increases for high-stage and high-grade tumors, especially for primary tumors [10]. It has been proposed that a bladder field defect may be causing the high frequency of tumor recurrences in bladder cancer patients [11]. Several molecular changes have been shown to be present in normal appearing areas of the urothelium in patients with bladder cancer [12], [13], and recently an epigenetic field defect was described [14].

Epigenetics is the study of mitotically and/or meiotically heritable changes in gene expression that cannot be explained by changes in DNA sequence [15]. DNA methylation is a well-studied epigenetic mechanism involved in normal processes like development, genomic imprinting, and X-chromosome inactivation [16]–[18]. Alterations in DNA methylation have been associated with several human pathologic disorders including cancer [19], and transcriptional inactivation by aberrant hypermethylation is a well-established mechanism for gene silencing in bladder cancers [20]–[23]. Identification of DNA methylation markers for detection of bladder cancer has been ongoing for some years. Several studies have reported high sensitivities and specificities for these markers, making them potentially useful as diagnostic markers for bladder cancer [24]–[31]. In a previous study we identified a number of DNA methylation markers (*EOMES*, *HOXA9*, *POU4F2,* and *ZNF154*) that showed potential diagnostic value in urine specimens from BC patients [26].

We now validated the diagnostic and prognostic value of these biomarkers together with earlier reported *TWIST1* [25] and *VIM* [30] in urine samples. Initially, to validate the significance of the selected methylation markers, and to determine of the marker cut-off values, we compared the first urine sample from each patient with NMIBC to urine samples from healthy individuals. Following, we evaluated the diagnostic and prognostic value of the markers for early detection of tumor recurrence using urine samples obtained at later follow-up visits.

## Materials and Methods

### Ethics Statement

Informed written consent was obtained from all patients, and research protocols were approved by the Central Denmark Region Committees on Biomedical Research Ethics.

### Patient Material

A total of 652 voided urine samples were collected at the Department of Urology at Aarhus University Hospital from 390 bladder cancer patients and 47 individuals with benign prostatic hyperplasia or bladder stones, but no history of bladder cancer (control individuals). From these we excluded 227 samples, because the DNA amount was below our threshold (Suppl. Table 1), which left 425 samples (390 samples from 184 BC patients and 35 from control individuals) (Table 1 and Suppl. Figure 1). Ten to fifty mL urine was collected at regular follow-up visits. Urine specimens were collected immediately before cystoscopy; cells were sedimented by centrifugation, and frozen at −80°C. The tumors were staged according to the TNM system [32] and graded according to Bergkvist [33]. Fifteen of the control individuals were stix positive for nitrite in the urine indicating bacterial infection. Patient treatment and follow-up were performed in accordance with the guidelines of the European Association of Urology [34].

**Table 1:** Demographic and clinical characteristics of bladder cancer patients and control individuals.

Characteristics	Control individuals		
**Individuals with no history of BC (controls)**	35		
Gender, n (%)			
Male	30 (86)		
Female	5 (14)		
Age, mean (min-max)	70 (35–88)		
Nitrite test, n (%)			
Positive	15 (43)		
Negative	20 (57)		
**Characteristics**	**All patients – first visit**	**Patients with recurrent tumor** **at control visit**	**Patients without tumor at control visit**
**Bladder cancer patients**	184	101^c^	57^c^
Samples collected	184	139	67
Primary cases	44		
Recurrent cases	140	139	
Gender, n (%)			
Male	148 (81)	106 (76)	58 (87)
Female	36(19)	33 (24)	9 (13)
Age, mean (min-max)	69 (33–89)	71 (43–89)	69 (49–86)
Ta	69 (33–85)	70 (43–87)	
T1	70 (42–89)	74 (43–89)	
CIS	71 (67–74)	73 (66–81)	
T2-4	0	71 (43–83)	
Pathological stage, n (%)			
Ta	132 (72)	92 (66)	
T1	50 (27)	29 (21)	
CIS	2 (1)	5 (4)	
T2-4	0	13 (9)	
Grade, n (%)^b^			
I	17 (9)	12 (9)	
II	74 (40)	55 (40)	
III	93 (51)	71 (51)	
Nitrite test, n (%)			
Positive	16 (9)	13 (9)	7 (10)
Negative	163 (89)	121 (87)	57 (85)
N/A^a^	5 (3)	5 (4)	3 (5)
Tumor cells in urine^d^, n (%)			
Positive	119 (65)	87 (63)	22 (33)
Negative	28 (15)	25 (18)	24 (36)
N/A	37 (20)	27 (19)	21 (31)

a N/A Not available.

b Bergkvist.

c Of the 184 patients, 26 were lost for follow-up.

d The presence of tumor cells in the urine was determined by urine cytology.

Demographic and clinical characteristics of bladder cancer patients and control individuals from whom urine specimens were collected and methylation analysis performed. Histology was used as the gold standard for the diagnosis of bladder tumors.

### DNA Extraction and Bisulfite Modification

DNA was extracted with the QIAsymphony Virus/Bacteria Midi kit (96) (Qiagen) using the QIAsymphony® SP instrument and employing the Complex800_V5_DSP protocol. Five hundred nanograms of DNA was bisulfite modified using EZ-96 DNA methylation D5004 (Zymo Research) according to the manufacturer’s recommendations and eluted in 60 µl of elution buffer and stored at −20°C until use.

### Real-time Quantitative Methylation-specific Polymerase Chain Reaction (MethyLight)

Methylation analysis was performed using methyLight [35]. Primers and probes for the six genes of interest were designed to include eight to ten CpG dinucleotides (Suppl. Table 2). For normalization of DNA input material, we used the ALU-C4 repeat element sequence [36]. qPCR amplifications were carried out with the TaqMan Universal PCR Master Mix No AmpErase (Applied Biosystems) according to the manufacturer’s instructions in duplicates using 2 µl (5 ng) of bisulfite-modified DNA in a final volume of 5 µl in 384-well plates on an ABI 7900 HT Fast Real Time PCR System (Applied Biosystems). If duplicates were inconsistent, one replicate being positive for methylation and one negative for methylation, the analysis was repeated. The sample was excluded with regard to that specific marker if another inconsistent result was obtained. Amplification protocols are listed in suppl. Table 2. Amplification data were analyzed by sequence detection system (SDS 2.4, Applied Biosystems). Each plate included a serial dilution (25–0.04ng) of fully methylated DNA: CpGenome™ Universal Methylated DNA) (Millipore) with the gene of interest and ALU-C4, several no template controls (NTC) wells, 5 nanograms of a methylated control sample [CpGenome™ Universal Methylated DNA (Millipore)], and unmethylated sample consisting of whole-genome amplified DNA from peripheral blood DNA. The percentage of methylated reference (PMR) was calculated for each sample according to the equation: 100×[(gene-x copy value) sample/(ALU-C4 copy value) sample]/[(gene-x copy value) Universal Methylated DNA/(ALU-C4copy value) Universal Methylated DNA].

### Statistical Analysis

Stata 11 (Statacorp, Texas, USA) was used for all statistical calculations. Two-tailed tests were considered statistically significant if P<0.05. Methylation differences were evaluated by nonparametric Wilcoxon-Mann-Whitney test. Fisher’s exact test was used for analyzing dichotomous variables. The exact $x^2 $test was used for analyzing associations between clinic-pathological parameters with two or more categories. Correlations of the methylation levels of the markers were calculated with Spearman correlation coefficients. A ROC curve was made for each marker and combinations of markers by plotting sensitivity against (1-specificity) and the area under the curve (AUC) was calculated. Log-Rank tests were applied to evaluate equality of survival and Kaplan-Meier survival plots were used for visualization. Univariate Cox regression analysis was used to analyze associations of age, gender, stage, grade, multiplicity, and CIS with recurrence-free survival.

## Results

Our analysis was divided into two parts: 1) to establish the cutoff level of the methylation markers and to demonstrate the significance of the selected markers we analyzed the first urine sample from each patient and compared to control urine samples from healthy individuals; 2) using the determined marker cutoff levels we then validated the diagnostic and prognostic value of the methylation markers in urine samples taken during patient surveillance.

### Establishment of Test Cut-off levels and Initial Validation of Marker Significance

Initially, we defined the cut-off levels by the mean methylation level of each marker +2x standard deviation of the methylation level in urine samples from 35 control individuals (only samples with methylation values above zero were included). Cut-off levels (PMR values – see materials and methods) used to dichotomize the methylation markers were: *EOMES* = 0.348, *HOXA9* = 0.077, *POU4F2* = 0.371, *TWIST1* = 0.405, *VIM = *0.368, and *ZNF154* = 1.51. Other cut-off levels (mean +1xSD and +3xSD) based on methylation levels in control urine samples were initially considered (results not shown). To validate the significance of the markers for bladder cancer diagnosis we compared the first urine sample from 184 patients diagnosed with NMIB to urine samples from 35 control individuals (Table 1). All six markers were highly significantly hyper-methylated in the urine from patients with NMIBC compared to urine from healthy individuals (Mann-Whitney, P<0.0001) (Table 2). Better sensitivities of the markers were observed when analyzing urine samples from patients with an incident tumor compared to urine from patients with a recurrent tumor (Suppl. Table 3). No association was observed between the individual markers and tumor stage, but *EOMES*, *POU4F2*, *VIM*, and *ZNF154* were more methylated in grade III lesions compared to grade I lesions (Fisher’s exact test, P≤0.048) (Suppl. Table 4). *EOMES*, *POU4F2,* and *ZNF154* were less methylated in tumors with a size below 3 cm. (Fisher’s exact test, P≤0.047) (Suppl. Table 4).

**Table 2:** Diagnostic significance of the urinary markers.

Gene	Sensitivity, % (pos./total^a^)	Specificity, % (neg./total)	AUC (95% CI)	PPV^b^, %	NPV^c^, %	P value^d^
***EOMES***	88 (160/182)	97 (34/35)	0.96 (0.94–0.99)	99	61	**<0.0001**
***HOXA9***	82 (141/173)	100 (35/35)	0.91 (0.88–0.94)	100	52	**<0.0001**
***POU4F2***	85 (154/182)	94 (33/35)	0.94 (0.91–0.97)	99	54	**<0.0001**
***TWIST1***	88 (159/180)	100 (35/35)	0.94 (0.92–0.97)	100	63	**<0.0001**
***VIM***	89 (159/179)	100 (35/35)	0.97 (0.94–0.99)	100	64	**<0.0001**
***ZNF154***	87 (160/184)	100 (35/35)	0.95 (0.93–0.97)	100	59	**<0.0001**
**Cytology**	81 (119/147)	N/A^e^	N/A	100	N/A	N/A

a Some urine samples provided inconclusive results for some markers.

b Positive predictive value.

c Negative predictive value.

d Mann-Whitney *U* test.

e Not available.

Diagnostic significance of the urinary markers *EOMES*, *HOXA9*, *POU4F2*, *TWIST1, VIM,* and *ZNF154*, when comparing urine samples from 184 patients with NMIBC to urine from healthy individuals.

### Detection of Recurrences by Methylation Markers

We validated the clinical usefulness of the markers for bladder cancer surveillance. We stratified our analysis to only include patients that initially showed hypermethylation of one or more methylation markers. Depending on the marker studied, 11–18% of patients showed no methylation in the first tumor and was therefore not included. This restricted the analysis to 158 patients and 206 urine samples from the follow-up visits; 139 urine samples were from patients with a recurrent bladder tumor and 67 urine samples were from patients with no tumor recurrence (Table 1). Employing the cut-points determined initially using the urine from healthy individuals; we obtained sensitivities in the range from 87% to 94%, and specificities in the range from 28% to 47% (Table 3). In comparison, the sensitivity of cytology was 77% and the specificity was 60%. We observed no significant associations between methylation levels and clinicopathologic variables for this patient cohort with recurrent tumors (Suppl. Table 5).

**Table 3:** Diagnostic significance of the urinary markers for surveillance of bladder cancer.

Gene	Sensitivity, % (pos./total^a^)	Specificity, % (neg./total^a^)	AUC (95% CI)	PPV, %	NPV, %	P value^b^
***EOMES***	94 (116/124)	39 (24/61)	0.78 (0.71–0.85)	76	75	**<0.0001**
***HOXA9***	92 (108/117)	38 (18/48)	0.70 (0.61–0.80)	78	67	**<0.0001**
***POU4F2***	87 (104/120)	47 (28/60)	0.75 (0.68–0.83)	76	64	**<0.0001**
***TWIST1***	89 (113/127)	28 (17/60)	0.71 (0.63–0.80)	72	55	**<0.0001**
***VIM***	90 (113/126)	43 (24/56)	0.72 (0.63–0.81)	78	65	**<0.0001**
***ZNF154***	93 (115/123)	47 (29/62)	0.78 (0.71–0.86)	78	78	**<0.0001**
**Cytology**	77 (88/115)	60 (35/58)	0.68 (0.61–0.76)	79	56	**<0.0001**

a Some urine samples provided inconclusive results for some markers.

b Mann-Whitney *U* test.

Diagnostic significance of the urinary markers *EOMES*, *HOXA9*, *POU4F2*, *TWIST1, VIM,* and *ZNF154*, when comparing urine samples from patients with NMIBC to urine samples from bladder cancer patients with no recurrence and using DNA collected at control visits in patients with a methylation positive first tumor. Histology was used as the gold standard for the diagnosis of bladder tumors.

The molecular tests may have a higher sensitivity compared to the gold standard cystoscopy. To address this we therefore used cystoscopy results from a 12 months period after the urine was sampled. We found many of the samples formerly classified as false positives to be true positives; they simply had a positive lead time compared to cystoscopy. The sensitivity obtained ranged from 88% to 94%, and the specificity ranged from 43% to 67% (Table 4). Including tumors diagnosed during a 12 month follow-up period and combining two markers with the requirement that both markers were positive for a positive test result the sensitivity decreased (range: 86–93%), while the specificity increased (range: 50–73%). If just one of two markers should be positive, the sensitivity increased (range: 92–98%), while the specificity decreased (range: 29–60%) (Suppl. Table 6 and Suppl. Table 7, respectively). Overall, combinations of markers did not improve both sensitivity and specificity of the tests.

**Table 4:** Diagnostic significance of the urinary markers for surveillance of bladder cancer when including a 12-months follow-up period.

Gene	Sensitivity, % (pos./total^a^)	Specificity, % (neg./total^a^)	AUC (95% CI)	PPV, %	NPV, %	P value^b^
***EOMES***	94 (133/141)	55 (24/44)	0.85 (0.77–0.92)	87	75	**<0.0001**
***HOXA9***	93 (123/132)	55 (18/33)	0.78 (0.68–0.89)	89	67	**<0.0001**
***POU4F2***	88 (120/136)	64 (28/44)	0.80 (0.72–0.89)	88	64	**<0.0001**
***TWIST1***	90 (133/147)	43 (17/40)	0.76 (0.66–0.86)	85	55	**<0.0001**
***VIM***	90 (129/143)	59 (23/39)	0.78 (0.68–0.89)	89	62	**<0.0001**
***ZNF154***	94 (134/142)	67 (29/43)	0.83 (0.74–0.92)	91	78	**<0.0001**
**Cytology**	79 (99/126)	74 (35/47)	0.77 (0.69–0.84)	89	56	**<0.0001**

a Some urine samples provided inconclusive results for some markers.

b Mann-Whitney *U* test.

Diagnostic significance of the urinary markers *EOMES*, *HOXA9*, *POU4F2*, *TWIST1, VIM,* and *ZNF154*, when comparing urine samples from patients with NMIBC to urine samples from bladder cancer patients with no recurrence and using DNA collected at control visits in patients with a methylation positive first tumor. Tumors diagnosed during a 12 month follow-up period were included. Histology was used as the gold standard for the diagnosis of bladder tumors.

### Prognostic Value of Methylation Markers for Predicting Later Recurrences

**Figure 1:**
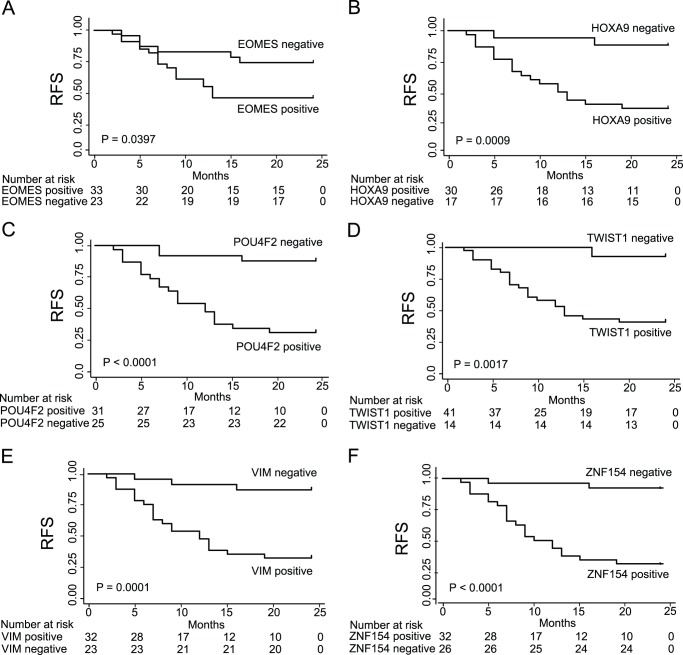
Kaplan-Meier plot of the time to recurrence. DNA methylation is associated with subsequent tumor recurrence within 24 months for patients without tumor but with methylation-positive urine samples. Kaplan-Meier plots of recurrence-free survival as a function of dichotomized methylation levels for *EOMES* (P = 0.0397) (A), *HOXA9* (P = 0.0009) (B), *POU4F2* (P<0.0001) (C), *TWIST1* (P = 0.0017) (D), *VIM* (P = 0.0001) (E), and *ZNF154* (P<0.0001) (F).

To address the prognostic value of the methylation markers we analyzed the urine samples from patients at visits where no tumors were diagnosed using cystoscopy. For all markers we found that a positive marker at a tumor-negative visit was significantly associated with later tumor recurrence during 24- and 60-month follow-up periods (Log-Rank test, P≤0.04) (Figure 1 and Suppl. Figure 3). The most significant differences in the 24-month time-frame were observed for *POU4F2* and *ZNF154* (Log-Rank test, P<0.0001) where only 12% (3/25) and 8% (2/26) with no methylation experienced a recurrence within 2 years, respectively. For the methylation-positive samples the percentage of patients with later recurrence was 68% (21/31) for *POU4F2* and 63% (20/32) for *ZNF154*. Univariate Cox regression analysis showed that *HOXA9* (HR (95% CI) = 7.8 (1.8–33.7)), *POU4F2* (HR (95% CI) = 8.5 (2.5–28.5)), *TWIST1* (HR (95% CI) = 12.0 (1.6–88.6)), *VIM* (HR (95% CI) = 8.0 (2.4–26.8)), and *ZNF154* (HR (95% CI) = 13.9 (3.3–59.7)) were significantly associated with recurrence-free survival. Age, gender, previous stage, previous grade, previous multiplicity, and previous CIS were not significantly associated with recurrence-free survival (P>0.05). Consequently, the presence of an altered methylation of DNA in urine seemed to be strongly related to the prognosis.

### Identification of Patients with a Possible Epigenetic Field Defect

If the methylation of the biomarkers was confined to malignant cells, we should only detect the markers in urine when a tumor was present, or occurring within a foreseeable future depending on the growth rate of the tumor. However, our results showed that even high urinary levels of methylation could be present at visits without recurrences (Suppl. Figure 2, patients C and D; Suppl. Figure 3). We could identify one group of 15 patients (31%) in which methylation was present in urine at the first visit and continued to be present in the urine samples taken at later follow-up visits, although no tumor occurred. As an example, one methylation-positive patient with the most prolonged follow-up was diagnosed with CIS after 118 months, but had no lesions in between. The 15 patients with a field defect, but no recurrence, have a significant lower number of tumors at previous visits (P = 0.02) compared to patients with disease recurrence.

**Figure 2:**
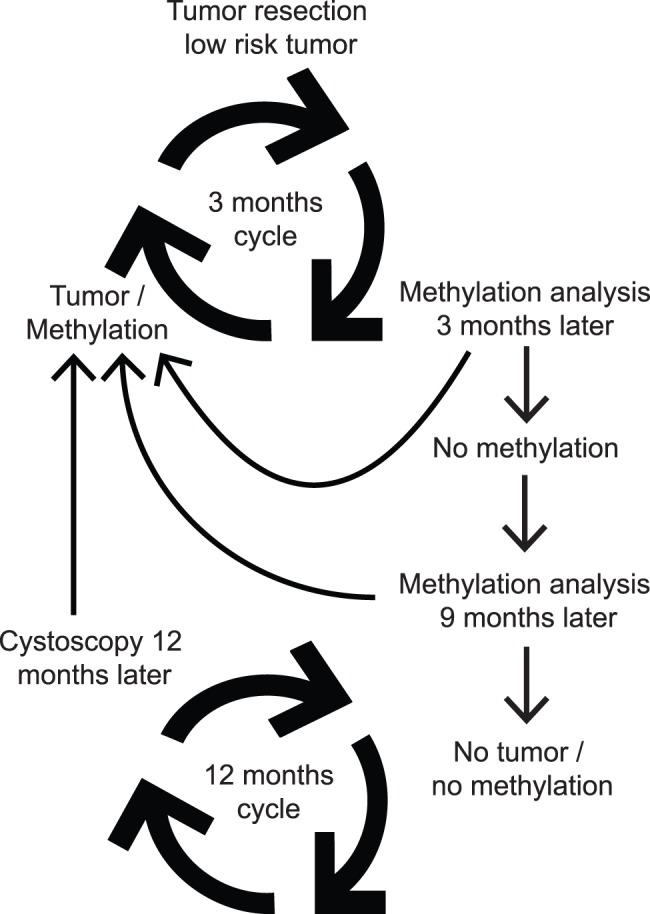
Follow-up model for low-risk NMIBC applying methylation markers. Modified from Hermann GG et al. (http://skejby.net/Webudgaven/DaBlaCa2010.htm.).

## Discussion

In this study we performed an independent validation of *EOMES*, *HOXA9*, *POU4F2*, *TWIST1*, *VIM*, and *ZNF154* methylation for the urinary diagnosis in bladder cancer surveillance. We obtained sensitivities in the range 87%–93% and specificities in the range 28%–47%. When including tumors resected in a 12-month follow-up period we obtained sensitivities in the range 88%–94% and specificities in the range 43%–67%. Univariate Cox regression analysis showed that the methylation markers predicted future recurrences with hazard ratios in the range from 7.8 to 13.9 (P≤0.015). Previous stage, grade, multiplicity and CIS were not significantly associated with tumor recurrence. Interestingly, we also observed that some bladder cancer patients without recurrence still maintained aberrant urinary methylation that distinguished them from control individuals. We believe that this may be caused by a general epigenetic bladder mucosa alteration.

The controls applied in this study were from patients with benign prostatic hyperplasia (BPH) or bladder stones and 43% of the controls were nitrite positive, indicating bladder infections. Some of the cells in the urine of the controls may originate from the hyperplasia, or may be immunological or bacterial cells. Similar control samples were applied when the markers were initially investigated as markers of bladder cancer, suggesting that the methylation frequency of the selected markers is low in these cell types [25], [26], [30]. Cells from the prostate or from a bladder infection may still bias the obtained results by dilution of the tumor cells. This could lead to false negative test results. Bacterial infections are much less common and in our study infections were not significantly associated with marker methylation, indicating that infections did not influence the marker sensitivity (Suppl. Table 4).

It is noteworthy that only 9% of the recurrences are grade I. Therefore the calculated sensitivities of the markers may be higher compared to sensitivities that would be obtained from a consecutive series of low-risk patients with a much higher number of grade I tumors.

One of the main challenges using urinary markers is getting sufficient tumor cells, and while MethyLight is a very sensitive method we still had to exclude 227 (35%) samples from the study due to insufficient amounts of DNA. We observed that if we included samples with less than 5 ng of DNA as template in the analysis, the sensitivity decreased, and for patients under surveillance the specificity increased. By excluding patients based on the amount of DNA extracted from the voided urine samples, we maintain the integrity of the MethyLight assay at the cost of introducing a bias where especially patients with stage Ta grade I tumors are excluded, as grade I lesions exfoliate the smallest number of cells [37] (Fisher’s exact test, P<0.05) (Suppl. Table 1). Recently, a study by Zuiverloon et al. reported an increase in the sensitivity of the *FGFR3* mutation as a marker for tumor recurrence from 75% to 100% when the volume of urine used for the test was increased [38]. By increasing the volume of collected urine from the current 10 to 50 mL, fewer samples will have to be excluded [39], [40]. Other possibilities to improve the assay for detecting methylation may include analyzing two or more samples separately or to use another technology than Methylight (e.g. Nested PCR).

The methylation marker test may be complemented by *FGFR3* mutation analysis. *FGFR3* mutations are present in about 70%–80% of low-grade NMIBC, and mutations in the *FGFR3* gene have been shown to be detectable in DNA from voided urine [41], [42]. Similar to the methylation analysis, the *FGFR3* analysis is based on DNA, and the primary visit can be used to stratify for the presence of the mutation. Recent studies with *FGFR3* mutations alone to detect NMIBC recurrences report a sensitivity of 58% for concomitant recurrences. The sensitivity increased when 12 months of follow-up were included [43]. The combination of *FGFR3* mutations and methylation markers has been tested by Serizawa et al., and they observed an increase in sensitivity of DNA from voided urine when combining methylation markers and *FGFR3* mutations [44]. It is noteworthy that they observed an inverse correlation between hypermethylation and *FGFR3* mutations.

The fact that we find no associations between methylation and clinicopathologic variables when using only urine from patients with recurrent tumors is intriguing, but it may be that the lower sensitivity observed in low-grade tumors is not only caused by few exfoliated tumor cells in the urine, but rather that the hypermethylation is not present in the tumor or in the surrounding urothelium, and by stratifying for methylation at an earlier visit these patients are excluded. It is possible that patients with no methylation are to be considered as a distinct group of bladder tumors. Finally, the lack of association between methylation and clinic-pathologic variables may also be a consequence of the relatively small data set.

The methylation markers have specificity values in the range 43% to 67%, which is low considering that they all have between 94%–100% tumor specificity when compared to control individuals. An equivalent low specificity was observed in a study investigating the mRNA levels of hTERT, SENP1, PPP1CA, and MCM5 in urine obtained from patients during disease surveillance [45]. Low specificity was also observed by Zuiverloon et al. when studying *FGFR3* mutations to detect bladder cancer recurrences [38]. Furthermore, low specificity of methylation markers was previously reported elsewhere by two studies aimed at detecting NMIBC recurrences [46], [47].

The methylation observed in urine samples from cystoscopy-negative visits may have several sources, (i) a small tumor not detected by cystoscopy, (ii) residual tumor cells at site of the resection, or (iii) it may be a symptom of epigenetically changed urothelial cells present in the urothelium surrounding the tumor or at other places (field defect) that have no phenotype to distinguish them from normal urothelial cells – an epigenetic urothelial methylator phenotype [14]. Some of the false positive samples with a recurrence within 12 months after cystoscopy are most likely caused by missed tumors or residual tumor cells, but for the rest of the false positive samples this explanation is unlikely and our results correspond well with the presence of a bladder field defect. Recent data support the notion that there is a DNA hypermethylation field defect in bladders from BC patients where the normal-appearing tissue contains widespread DNA methylation [14]. Wolff and co-workers suggest that the aberrant methylation is not due to clonal expansion, but instead is caused by generalized epigenetic alteration in the urothelium across the bladder. It has been suggested that this widespread field of aberrant methylated normal-appearing urothelium may be the origin of the high recurrence rate in bladder cancer [14].

With the discovery of methylation markers with very high sensitivity, implementation of methylation markers in the surveillance of patients with low-grade NMIBC seems likely. At the time of diagnosis, the methylation level of each methylation marker has to be established before the marker can be applied for surveillance. In advance of the next control visit, the patient may supply a urine sample for the analysis, and three possible outcomes exist: i) if the test is positive the patient will have a cystoscopy done, and in 67 patients out of 100 a recurrent tumor will be found, whereas the remaining 33 patients will not have a tumor recurrence. This is not a major problem, as the false positive patients would have had a cystoscopy done in any case; ii) a negative test will allow the patients to skip the current cystoscopy. With the current performance of the analysis, 94 out of 100 BC patients with a tumor recurrence are correctly diagnosed and six patients are wrongly diagnosed as not having a tumor recurrence. This amount of false negatives is comparable with HAL-guided cystoscopy and better than white light cystoscopy, where 20 patients can be expected to be wrongly diagnosed as not having a tumor recurrence [7]–[9]. However, in this study the *ZNF154* marker failed to diagnose two muscle-invasive bladder cancers that progressed from two T1 grade III tumors. This would not be acceptable in a clinical setting, and indicates that patients with a high risk of progression (e.g. all T1, grade III tumors, or CIS) have to continue with the regular treatment regimen. Another option could be to use a more conservative cut-off. By defining the cut-off values as the mean plus 1x standard deviation of the methylation level, the two muscle invasive tumors would not have been missed; iii) a urine sample with insufficient DNA should lead to continuation of the original treatment regimen. In all three situations the patient will supply a new urine sample before the next control visit that will determine whether or not the patient must have a cystoscopy performed. The methylation markers may also increase the detection rate of CIS lesions, as a positive test with a negative cystoscopy could then be followed by another cystoscopy including HAL.

In conclusion, by applying a very sensitive and semi-quantitative methodology for detecting bladder cancer recurrences and stratifying for methylation status of the initial tumor, we have shown that using a single marker (*ZNF154*) we can detect a concomitant tumor recurrence with a sensitivity of 94% and a specificity of 67%. According to the EAU Guidelines on NMIBC, the current treatment regime for low-risk NMIBC patients is cystoscopy at 3 and 12 months following TUR and then each year for an additional 4 years [48]. This study suggests that methylation markers can be utilized as markers of bladder cancer recurrence to reduce the number of cystoscopies in low-risk patients with no concomitant tumor (Figure 2) and consequently improve the quality of life for the patients as well as decrease health care expenditure.

## Supporting Information

Figure S1 **Flow chart illustrating the flow of samples during the course of the study.**
(EPS)

Figure S2 **Examples of methylation levels at first visit and two subsequent visits for 5 patients with or without recurrences.** Patients with two subsequent recurrences (A and B). Patient with no recurrences at first control visit, but recurrence at a later control visits (C and D). A patient with no subsequent recurrences (E). PMR is the percentage of relative reference. A value of 0% means no methylation and a value of 100% means fully methylated. A dark bar represents a visit with concomitant tumor and a gray bar represents a visit with no tumor.
(EPS)

Figure S3 **Kaplan-Meier plot of the time to recurrence.** DNA methylation is associated with subsequent tumor recurrence within 60 months for patients without tumor but with methylation positive urine samples. Kaplan-Meier plots of recurrence-free survival as a function of dichotomized methylation levels for *EOMES* (P = 0.0254) (A), *HOXA9* (P = 0.0024) (B), *POU4F2* (P = 0.0001) (C), *TWIST1* (P = 0.0034) (D), and *VIM* (P = 0.0001) (E), and *ZNF154* (P<0.0001) (F).
(EPS)

Table S1 **Excluded samples.**
(DOCX)

Table S2 **Primer and probe sequences for real-time PCR.**
(DOCX)

Table S3 **Performance of the urinary markers on the first urine samples.**
(DOC)

Table S4 **Associations between methylation markers and clinicopathologic parameters.**
(DOC)

Table S5 **Associations between methylation markers and clinicopathologic parameters for patients under surveillance.**
(DOC)

Table S6 **Diagnostic significance of two markers both positive for methylation for a positive test result.**
(DOCX)

Table S7 **Diagnostic significance of two markers with one positive for methylation for a positive test result.**
(DOCX)
