# Supplementary material for: Diagnosis of Bladder Cancer Recurrence Based on Urinary Levels of EOMES, HOXA9, POU4F2, TWIST1, VIM, and ZNF154 Hypermethylation
Source: PLoS One. 2012 Oct 3;7(10):e46297. doi: 10.1371/journal.pone.0046297 (PMC3463582; doi:10.1371/journal.pone.0046297)
Supplement: Table S1 — Excluded samples. (DOCX) [file pone.0046297.s004.docx]

**Table S1.** Excluded samples.

| **Characteristics** | **Control individuals** |  |  |
| --- | --- | --- | --- |
| **Individual with no history of BC** | 12 |  |  |
| Gender, n (%) |  |  |  |
| Male | 11 (92) |  |  |
| Female | 1 (8) |  |  |
| Age, mean (min-max) | 64 (51-80) |  |  |
| Nitrite test, n (%) |  |  |  |
| Positive | 1 (8) |  |  |
| Negative | 11 (92) |  |  |
| **Characteristics** | **All patients – first visit** | **Patients with recurrent tumor at control visit** | **Patients without tumor at control visit** |
| **Bladder cancer patients** | 80 | 63 | 49 |
| Samples collected | 80 | 75 | 60 |
| Primary cases | 21 |  |  |
| Recurrent cases | 59 | 75 |  |
| Gender, n (%) |  |  |  |
| Male | 61 (76) | 59 (79) | 55 (92) |
| Female | 19 (24) | 16 (21) | 5 (8) |
| Age, mean (min-max) | 67 (33-83) | 70 (34-85) | 68 (43-84) |
| Ta | 67 (33-83) | 70 (34-85) |  |
| T1 | 69 (56-80) | 73 (65-82) |  |
| CIS | 67 (67-67) | 68 (57-73) |  |
| T2-4 | 0 | 72 (69-74) |  |
| Pathological stage, n (%) |  |  |  |
| Ta | 66 (83) | 57 (76) |  |
| T1 | 13 (16) | 11 (15) |  |
| CIS | 1 (1) | 5 (7) |  |
| T2-4 | 0 | 2 (3) |  |
| Grade, n (%)^a^ |  |  |  |
| I | 16 (20) | 14 (19) |  |
| II | 36 (45) | 34 (45) |  |
| III | 28 (35) | 27 (36) |  |
| Nitrite test, n (%) |  |  |  |
| Positive | 3 (4) | 4 (5) | 2 (3) |
| Negative | 75 (94) | 66 (88) | 57 (95) |
| N/A^b^ | 2 (3) | 5 (7) | 1 (2) |
| Tumor cells in urine, n (%) |  |  |  |
| Positive | 41 (51) | 31 (41) | 18 (30) |
| Negative | 33 (41) | 32 (43) | 27 (45) |
| N/A | 6 (8) | 12 (16) | 15 (25) |

^a^ Bergkvist

^b^ Not available

Demographic and clinical characteristics of bladder cancer patients and control individuals from whom urine specimens were collected, but without enough DNA for methylation analysis. Histology was used as the gold standard for the diagnosis of bladder tumors.
