# Supplementary material for: Diagnosis of Bladder Cancer Recurrence Based on Urinary Levels of EOMES, HOXA9, POU4F2, TWIST1, VIM, and ZNF154 Hypermethylation
Source: PLoS One. 2012 Oct 3;7(10):e46297. doi: 10.1371/journal.pone.0046297 (PMC3463582; doi:10.1371/journal.pone.0046297)
Supplement: Table S2 — Primer and probe sequences for real-time PCR. (DOCX) [file pone.0046297.s005.docx]

Table S2. Primer and probe sequences for real-time PCR.

| Gene | **Sense primer (5´to 3´)** | | **Antisense primer (5´to 3´)** | **Probe (5´to 3´)** | **Amplification protocols** |
| --- | --- | --- | --- | --- | --- |
| ***ALU-C4*** | GGTTAGGTATAGTGGTTTATATTTGTAATTTTAGTA | ATTAACTAAACTAATCTTAAACTCCTAACCTCA | | CCTACCTTAACCTCCC | 95°C 10’, (95°C 15’‘, 60°C 1‘) x45 |
| ***EOMES*** | GGTTGGGGAAGTAGAGTTTCGAT | ATAAACAATTACAAACGCCGCCA | | CGCTCCGAAAACGCATTTTCCGACTA | 95°C 10’, (95°C 15’‘, 60°C 1‘) x45 |
| ***HOXA9*** | GTGGTTATTATCGTGTTTAGCGT | CCGATACCACCAAATTATTACATA | | TGGTTCGTTCGGTTCGATTTACGGA | 95°C 10’, (95°C 15’‘, 60°C 1‘) x45 |
| ***POU4F2*** | GTTGTGCGAAGTTGAGTTTATTC | CCGTTCAAACTAACAACAAAAACGA | | CGGATTTTGTACGTTTGATTTCGGTTAC | 95°C 10’, (95°C 15’‘, 60°C 1‘) x45 |
| ***TWIST1*** | GTTAGGGTTCGGGGGCGTTGTT | CCGTCGCCTTCCTCCGACGAA | | CGGCGGGGAAGGAAATCGTTTC | 95°C 10’, (95°C 15’‘, 60°C 1‘) x45 |
| ***VIM*** | TTCGGGAGTTAGTTCGCGTT | ACCGCCGAACATCCTACGA | | TCGTCGTTTAGGTTATCGT | 95°C 10’, (95°C 15’‘, 60°C 1‘) x45 |
| ***ZNF154*** | TTTATCGGATTAGAGATAGTAGAGCGT | TAACGTAAATCCCCCAAAACGACG | | AACGACGACTCCCCTCACGCCTT | 95°C 10’, (95°C 15’‘, 60°C 1‘) x45 |

TaqMan probe sequences, and amplification protocols for real-time quantitative methylation-specific polymerase chain reaction. All probes contain a 6FAM fluorophore at the 5’ end and a black hole quencher-1 (BHQ-1) at the 3’ end.
