# Supplementary material for: Diagnosis of Bladder Cancer Recurrence Based on Urinary Levels of EOMES, HOXA9, POU4F2, TWIST1, VIM, and ZNF154 Hypermethylation
Source: PLoS One. 2012 Oct 3;7(10):e46297. doi: 10.1371/journal.pone.0046297 (PMC3463582; doi:10.1371/journal.pone.0046297)
Supplement: Table S3 — Performance of the urinary markers on the first urine samples. (DOC) [file pone.0046297.s006.doc]

**Table S3. Performance of the urinary markers on the first urine samples.**

| **Gene** | **Sensitivity, % (pos. / total^a^)** | **Specificity, % (neg. / total)** | **AUC (95% CI)** | **PPV^b^,**  **%** | **NPV^c^, %** | **P value^d^** |
| --- | --- | --- | --- | --- | --- | --- |
| **First urine analyzed from an incident tumor visit** | | | | | | |
| ***EOMES*** | 95(42/44) | 97 (34/35) | 1.00 (0.99-1.00) | 98 | 94 | **<0.0001** |
| ***HOXA9*** | 86 (38/44) | 100 (35/35) | 0.92 (0.87-0.98) | 100 | 85 | **<0.0001** |
| ***POU4F2*** | 100 (43/43) | 94 (33/35) | 1.00 (1.00-1.00) | 96 | 100 | **<0.0001** |
| ***TWIST1*** | 93 (41/44) | 100 (35/35) | 0.98 (0.95-1.00) | 100 | 92 | **<0.0001** |
| ***VIM*** | 95 (42/44) | 100 (35/35) | 0.98 (0.95-1.00) | 100 | 95 | **<0.0001** |
| ***ZNF154*** | 98 (43/44) | 100 (35/35) | 0.99 (0.96-1.00) | 100 | 97 | **<0.0001** |
| **Cytology** | 87 (33/38) | N/A^e^ | N/A | 100 | N/A | N/A |
| **First urine analyzed from a recurrent tumor visit** | | | | | | |
| ***EOMES*** | 86 (118/138) | 97 (34/35) | 0.96 (0.94-0.99) | 99 | 63 | **<0.0001** |
| ***HOXA9*** | 80 (103/129) | 100 (35/35) | 0.91 (0.88-0.94) | 100 | 57 | **<0.0001** |
| ***POU4F2*** | 80 (111/139) | 94 (33/35) | 0.94 (0.91-0.97) | 98 | 54 | **<0.0001** |
| ***TWIST1*** | 87 (118/136) | 100 (35/35) | 0.94 (0.92-0.97) | 100 | 66 | **<0.0001** |
| ***VIM*** | 87 (117/135) | 100 (35/35) | 0.97 (0.94-0.99) | 100 | 66 | **<0.0001** |
| ***ZNF154*** | 84 (117/140) | 100 (35/35) | 0.95 (0.93-0.97) | 100 | 60 | **<0.0001** |
| **Cytology** | 79 (86/109) | N/A | N/A | 100 | N/A | N/A |

^a^ Some urine samples provided inconclusive results for some markers

^b^ Positive predictive value

^c^ Negative predictive value

^d^ Mann-Whitney *U* test

^e^ Not available

Performance of the urinary markers *EOMES, HOXA9, POU4F2, TWIST1, VIM,* and *ZNF154* using DNA from 184 for patients with NMIBC, divided into incident and recurrent cases, compared to 35 control individuals with no history of bladder cancer. The urines were collected shortly before cystoscopy. Histology was used as the gold standard for the diagnosis of bladder tumors.
