# Supplementary material for: Diagnosis of Bladder Cancer Recurrence Based on Urinary Levels of EOMES, HOXA9, POU4F2, TWIST1, VIM, and ZNF154 Hypermethylation
Source: PLoS One. 2012 Oct 3;7(10):e46297. doi: 10.1371/journal.pone.0046297 (PMC3463582; doi:10.1371/journal.pone.0046297)
Supplement: Table S4 — Associations between methylation markers and clinicopathologic parameters. (DOC) [file pone.0046297.s007.doc]

**Table S4.** Associations between methylation markers and clinicopathologic parameters.

|  |  | ***EOMES*** | ***HOXA9*** | ***POU4F2*** | ***TWIST1*** | ***VIM*** | ***ZNF154*** |
| --- | --- | --- | --- | --- | --- | --- | --- |
| **Stage** | pTa | 86% (112/130) | 80% (99/124) | 83% (108/130) | 85% (109/128) | 89% (113/127) | 84% (111/132) |
|  | pT1 | 92% (46/50) | 87% (41/47) | 88% (44/50) | 96% (48/50) | 88% (44/50) | 94% (47/50) |
|  | CIS | 100% (2/2) | 50% (1/1) | 100% (2/2) | 100% (2/2) | 100% (2/2) | 100% (2/2) |
|  | P value^a^ | 0.572 | 0.229 | 0.640 | 0.125 | 0.841 | 0.188 |
| **Grade** | I | 65% (11/17) | 80% (12/15) | 59% (10/17) | 71% (10/14) | 75% (12/16) | 71% (12/17) |
|  | II | 85% (61/72) | 80% (56/70) | 82% (60/73) | 90% (66/73) | 86% (60/70) | 77% (57/74) |
|  | III | 95% (88/93) | 83% (73/88) | 91% (84/92) | 89% (83/93) | 94% (87/93) | 98% (91/93) |
|  | P value | **0.002** | 0.837 | **0.004** | 0.132 | **0.048** | **< 0.001** |
| **Age, years** | ≤ 70 | 81% (76/94) | 77% (67/87) | 81% (76/94) | 87% (81/93) | 86% (77/90) | 84% (80/95) |
|  | > 70 | 95% (84/88) | 86% (74/86) | 90% (78/88) | 90% (78/87) | 92% (82/89) | 90% (80/89) |
|  | P value | **0.003** | 0.170 | 0.157 | 0.648 | 0.235 | 0.281 |
| **Tumor size, cm** | < 3 cm. | 85% (125/147) | 81% (112/139) | 81% (119/147) | 86% (125/145) | 87% (125/144) | 84% (125/149) |
|  | > 3 cm. | 100% (23/23) | 91% (21/23) | 100% (23/23) | 100% (23/23) | 96% (22/23) | 100% (23/23) |
|  | P value | **0.047** | 0.376 | **0.016** | 0.079 | 0.315 | **0.047** |
| **Cytology** | Positive | 96% (113/118) | 86% (99/115 | 90% (106/118) | 91% (108/119) | 94% (108/115) | 95% (113/119) |
|  | Negative | 64% (18/28) | 68% (17/25) | 64% (18/28) | 80% (20/25) | 64% (18/28) | 54% (15/28) |
|  | P value | **< 0.001** | **0.041** | **0.002** | 0.156 | **< 0.001** | **< 0.001** |
| **Stix, nitrite** | Positive | 81% (13/16) | 93% (14/15) | 81% (13/16) | 81% (13/16) | 80% (12/15) | 75% (12/16) |
|  | Negative | 88% (142/161) | 80 (123/153) | 85% (137/161) | 89% (142/159) | 89% (142/159) | 88% (144/163) |
|  | P value* | 0.426 | 0.309 | 0.715 | 0.400 | 0.386 | 0.131 |

^a^ Fisher’s exact test

Associations between methylation markers and stage, grade, age, tumor size, cytology, and nitrite in urine specimens using DNA collected from 184 patients with a NMIBC diagnosed by histology shortly after sampling. Methylation values were dichotomized as positive or negative according to the cut-off values.
