# Supplementary material for: Diagnosis of Bladder Cancer Recurrence Based on Urinary Levels of EOMES, HOXA9, POU4F2, TWIST1, VIM, and ZNF154 Hypermethylation
Source: PLoS One. 2012 Oct 3;7(10):e46297. doi: 10.1371/journal.pone.0046297 (PMC3463582; doi:10.1371/journal.pone.0046297)
Supplement: Table S5 — Associations between methylation markers and clinicopathologic parameters for patients under surveillance. (DOC) [file pone.0046297.s008.doc]

**Table S5.** Associations between methylation markers and clinicopathologic parameters for patients under surveillance.

|  |  | ***EOMES*** | ***HOXA9*** | ***POU4F2*** | ***TWIST1*** | ***VIM*** | ***ZNF154*** |
| --- | --- | --- | --- | --- | --- | --- | --- |
| **Stage** | pTa | 91% (74/81) | 93% (74/80) | 85% (67/79) | 87% (74/85) | 89% (72/81) | 95% (74/78) |
|  | pT1 | 96% (26/27) | 92% (22/24) | 93% (26/28) | 93% (26/28) | 93% (26/28) | 93% (26/28) |
|  | CIS | 100% (5/5) | 100% (3/3) | 67% (2/3) | 75% (3/4) | 75% (3/4) | 100% (5/5) |
|  | T2-4 | 100% (11/11) | 90% (9/10) | 90% (9/10) | 100% (10/10) | 92% (12/13) | 83% (10/12) |
|  | P value^a^ | 0.719 | 0.894 | 0.453 | 0.387 | 0.606 | 0.424 |
| **Grade** | I | 88% (7/8) | 91% (10/11) | 81% (9/11) | 82% (9/11) | 89% (8/9) | 100% (9/9) |
|  | II | 96% (48/50) | 93% (43/46) | 89% (42/47) | 88% (46/52) | 92% (46/50) | 94% (46/49) |
|  | III | 92% (61/66) | 92% (55/60) | 85% (53/62) | 91% (58/64) | 88% (59/67) | 92% (60/65) |
|  | P value | 0.398 | 1.000 | 0.725 | 0.642 | 0.657 | 1.000 |
| **Age, years** | ≤ 70 | 96(44/46) | 90% (38/42) | 86% (38/44) | 88% (44/50) | 93% (42/45) | 90% (43/48) |
|  | > 70 | 92% (72/78) | 93% (70/75) | 87% (66/76) | 90% (69/77) | 88% (71/81) | 96% (72/75) |
|  | P value | 0.709 | 0.720 | 1.000 | 0.779 | 0.376 | 0.260 |
| **Tumor size, cm** | < 3 cm. | 92% (94/102) | 91% (88/97) | 86% (85/99) | 89% (94/106) | 89% (92/103) | 93% (92/99) |
|  | > 3 cm. | 100% (12/12) | 100% (11/11) | 100% (11/11) | 100% (12/12) | 100% (12/12) | 100% (12/12) |
|  | P value | 0.597 | 0.594 | 0.353 | 0.609 | 0.602 | 1.000 |
| **Cytology** | Positive | 94% (77/82) | 93% (68/73) | 89% (67/75) | 87% (70/79) | 90% (73/81) | 95% (74/78) |
|  | Negative | 94% (17/18) | 95% (18/19) | 81% (17/21) | 90% (19/21) | 85% (17/20) | 85% (17/20) |
|  | P value | 1.000 | 1.000 | 0.289 | 1.000 | 0.452 | 0.148 |
| **Stix, nitrite** | Positive | 82% (9/11) | 100% (11/11) | 77% (10/13) | 85% (11/13) | 77% (10/13) | 83% (10/12) |
|  | Negative | 95% (104/110) | 92 (94/102) | 87% (90/103) | 89% (97/109) | 92% (100/109) | 94% (101/107) |
|  | P value | 0.156 | 1.000 | 0.386 | 0.645 | 0.118 | 0.185 |

^a^ Fisher’s exact test

Associations between methylation markers and stage, grade, age, tumor size, cytology, and nitrite in urine specimens using DNA collected from 101patients with a recurrent tumor diagnosed by histology shortly after sampling. Methylation values were dichotomized as positive or negative according to the cut-off values.
