# Supplementary material for: Diagnosis of Bladder Cancer Recurrence Based on Urinary Levels of EOMES, HOXA9, POU4F2, TWIST1, VIM, and ZNF154 Hypermethylation
Source: PLoS One. 2012 Oct 3;7(10):e46297. doi: 10.1371/journal.pone.0046297 (PMC3463582; doi:10.1371/journal.pone.0046297)
Supplement: Table S6 — Diagnostic significance of two markers both positive for methylation for a positive test result. (DOCX) [file pone.0046297.s009.docx]

**Table S6.** Diagnostic significance of two markers both positive for methylation.

| **Gene** | **Sensitivity, % (pos. / total^a^)** | **Specificity, % (neg. / total^a^)** |
| --- | --- | --- |
| ***EOMES* and *HOXA9*** | 89 (106/119) | 68 (21/31) |
| ***EOMES* and *POU4F2*** | 86 (110/128) | 73 (30/41) |
| ***EOMES* and *TWIST1*** | 89 (117/131) | 61 (23/38) |
| ***EOMES* and *VIM*** | 90 (119/132) | 71 (27/38) |
| ***EOMES* and *ZNF154*** | 91 (122/134) | 73 (29/40) |
| ***HOXA9* and *POU4F2*** | 87 (104/120) | 62 (18/29) |
| ***HOXA9* and *TWIST1*** | 87 (109/125) | 50 (15/30) |
| ***HOXA9* and *VIM*** | 89 (109/123) | 62 (18/29) |
| ***HOXA9* and *ZNF154*** | 93 (111/120) | 67 (20/30) |
| ***POU4F2* and *TWIST1*** | 86 (112/130) | 68 (27/40) |
| ***POU4F2* and *VIM*** | 89 (117/132) | 63 (24/38) |
| ***POU4F2* and *ZNF154*** | 88 (113/129) | 68 (27/40) |
| ***TWIST1* and *VIM*** | 89 (112/126) | 53 (18/34) |
| ***TWIST1* and *ZNF154*** | 90 (119/132) | 65 (24/37) |
| ***VIM* and *ZNF154*** | 91 (121/133) | 65 (24/37) |

^a^ Some urine samples provided inconclusive results for some markers.

Diagnostic significance of pairs of urinary markers when comparing urine samples from patients with NMIBC to urine samples from bladder cancer patients with no recurrence and using DNA collected at control visits in patients with a methylation positive first tumor. Only samples with conclusive results from both markers were included and both markers had to be positive for methylation for a positive test result. Tumors diagnosed during a 12 month follow-up period were included. Histology was used as the gold standard for the diagnosis of bladder tumors.
